# Leafy automata for higher-order concurrency

**DOI:** 10.1007/978-3-030-71995-1_10

**Published:** 2021-03-23

**Authors:** Alex Dixon, Ranko Lazić, Andrzej S. Murawski, Igor Walukiewicz

**Affiliations:** 8 University of Oxford, Oxford, UK; 9 Sorbonne Université - LIP6, Paris, France; 10 University of Warwick, Coventry, UK; 11 University of Warwick, Coventry, UK; 12 University of Oxford, Oxford, UK; 13 CNRS, Université de Bordeaux, Talence, France

**Keywords:** Finitary Idealized Concurrent Algol, Higher-Order Concurrency, Automata over Infinite Alphabets, Game Semantics

## Abstract

Finitary Idealized Concurrent Algol ($\mathsf {FICA}$) is a prototypical programming language combining functional, imperative, and concurrent computation. There exists a fully abstract game model of $\mathsf {FICA}$, which in principle can be used to prove equivalence and safety of $\mathsf {FICA}$ programs. Unfortunately, the problems are undecidable for the whole language, and only very rudimentary decidable sub-languages are known.

We propose leafy automata as a dedicated automata-theoretic formalism for representing the game semantics of $\mathsf {FICA}$. The automata use an infinite alphabet with a tree structure. We show that the game semantics of any $\mathsf {FICA}$ term can be represented by traces of a leafy automaton. Conversely, the traces of any leafy automaton can be represented by a $\mathsf {FICA}$ term. Because of the close match with $\mathsf {FICA}$, we view leafy automata as a promising starting point for finding decidable subclasses of the language and, more generally, to provide a new perspective on models of higher-order concurrent computation.

Moreover, we identify a fragment of $\mathsf {FICA}$ that is amenable to verification by translation into a particular class of leafy automata. Using a locality property of the latter class, where communication between levels is restricted and every other level is bounded, we show that their emptiness problem is decidable by reduction to Petri net reachability.

## 1 Introduction

Game semantics is a versatile paradigm for giving semantics to a wide spectrum of programming languages [3,35]. It is well-suited for studying the observational equivalence of programs and, more generally, the behaviour of a program in an arbitrary context. About 20 years ago, it was discovered that the game semantics of a program can sometimes be expressed by a finite automaton or another simple computational model [20]. This led to algorithmic uses of game semantics for program analysis and verification [1,15,21,5,27,26,28,34,16,17]. Thus far, these advances concerned mostly languages without concurrency.

In this work, we consider Finitary Idealized Concurrent Algol (FICA) and its fully abstract game semantics [22]. It is a call-by-name language with higher-order features, side-effects, and concurrency implemented by a parallel composition operator and semaphores. It is finitary since, as it is common in this context, base types are restricted to finite domains. Quite surprisingly, the game semantics of this language is arguably simpler than that for the language without concurrency. The challenge comes from algorithmic considerations.

Following the successful approach from the sequential case [20,37,33,36,11], the first step is to find an automaton model abstracting the phenomena appearing in the semantics. The second step is to obtain program fragments from structural restrictions on the automaton model. In this paper we take both steps.

We propose *leafy automata*: an automaton model working on nested data. Data are used to represent pointers in plays, while the nesting of data reflects structural dependencies in the use of pointers. Interestingly, the structural dependencies in plays boil down to imposing a tree structure on the data. We show a close correspondence between the automaton model and the game semantics of FICA. For every program, there is a leafy automaton whose traces (data words) represent precisely the plays in the semantics of the program (Theorem 3). Conversely, for every leafy automaton, there is a program whose semantics consists of plays representing the traces of the automaton (Theorem 5). (The latter result holds modulo a saturation condition we explain later.) This equivalence shows that leafy automata are a suitable model for studying decidability questions for FICA.

Not surprisingly, due to their close connection to FICA, leafy automata turn out to have an undecidable emptiness problem. We use the undecidability argument to identify the source, namely communication across several unbounded levels, i.e., levels in which nodes can produce an unbounded number of children during the lifetime of the automaton. To eliminate the problem, we introduce a restricted variant of leafy automata, called *local*, in which every other level is bounded and communication is allowed to cross only one unbounded node. Emptiness for such automata can be decided via reduction to a number of instances of Petri net reachability problem.

We also identify a fragment of FICA, dubbed *local* FICA (LFICA), which maps onto local leafy automata. It is based on restricting the distance between semaphore and variable declarations and their uses inside the term. This is a first non-rudimentary fragment of FICA for which some verification tasks are decidable. Overall, this makes it possible to use local leafy automata to analyse LFICA terms and decide associated verification tasks.

*Related work* Concurrency, even with only first-order recursion, leads to undecidability [39]. Intuitively, one can encode the intersection of languages of two pushdown automata. From the automata side, much research on decidable cases has concentrated on bounding interactions between stacks representing different threads of the program [38,30,4]. From the game semantics side, the only known decidable fragment of FICA is Syntactic Control of Concurrency (SCC) [23], which imposes bounds on the number of threads in which arguments can be used.

This restriction makes it possible to represent the game semantics of programs by finite automata. In our work, we propose automata models that correspond to unbounded interactions with arbitrary FICA contexts, and importantly that remains true also when we restrict the terms to LFICA. Leafy automata are a model of computation over an infinite alphabet. This area has been explored extensively, partly motivated by applications to database theory, notably XML [41]. In this context, nested data first appeared in [7], where the authors considered shuffle expressions as the defining formalism. Later on, data automata [9] and class memory automata [8] have been adapted to nested data in [14,12]. They are similar to leafy automata in that the automaton is allowed to access states related to previous uses of data values at various depths. What distinguishes leafy automata is that the lifetime of a data value is precisely defined and follows a question and answer discipline in correspondence with game semantics. Leafy automata also feature run-time "zero-tests", activated when reading answers.

For most models over nested data, the emptiness problem is undecidable. To achieve decidability, the authors in [14,12] relax the acceptance conditions so that the emptiness problem can eventually be recast as a coverability problem for a well-structured transition system. In [10], this result was used to show decidability of equivalence for a first-order (sequential) fragment of Reduced ML. On the other hand, in [7] the authors relax the order of letters in words, which leads to an analysis based on semi-linear sets. Both of these restrictions are too strong to permit the semantics of FICA, because of the game-semantic WAIT condition, which corresponds to waiting until all sub-processes terminate.

Another orthogonal strand of work on concurrent higher-order programs is based on higher-order recursion schemes [24,29]. Unlike FICA, they feature recursion but the computation is purely functional over a single atomic type $o$.

*Structure of the paper:* In the next two sections we recall FICA and its game semantics from [22]. The following sections introduce leafy automata (LA) and their local variant (LLA), where we also analyse the associated decision problems and, in particular, show that the non-emptiness problem for LLA is decidable. Subsequently, we give a translation from FICA to LA (and back) and define a fragment LFICA of FICA which can be translated into LLA. We will occasionally refer the reader to the full paper [18] which includes appendices with proof details and worked examples.

## 2 Finitary Idealized Concurrent Algol (FICA)

Idealized Concurrent Algol [22] is a paradigmatic language combining higher-order with imperative computation in the style of Reynolds [40], extended to concurrency with parallel composition (||) and binary semaphores. We consider its finitary variant FICA over the finite datatype $\{0,\ldots,max\}$ ($max \geq 0$) with loops but no recursion. Its types $\theta$ are generated by the grammar

$$\theta ::= \beta \mid \theta \rightarrow \theta \qquad\qquad \beta ::= \mathbf{com} \mid \mathbf{exp} \mid \mathbf{var} \mid \mathbf{sem}$$

$$\frac{}{\Gamma \vdash \mathbf{skip} : \mathbf{com}} \qquad \frac{}{\Gamma \vdash \mathbf{div}_\theta : \theta} \qquad \frac{}{\Gamma \vdash i : \mathbf{exp}} \qquad \frac{\Gamma \vdash M : \mathbf{exp}}{\Gamma \vdash \mathbf{op}(M) : \mathbf{exp}}$$

$$\frac{\Gamma \vdash M : \mathbf{com} \qquad \Gamma \vdash N : \beta}{\Gamma \vdash M; N : \beta} \qquad \frac{\Gamma \vdash M : \mathbf{com} \qquad \Gamma \vdash N : \mathbf{com}}{\Gamma \vdash M || N : \mathbf{com}}$$

$$\frac{\Gamma \vdash M : \mathbf{exp} \qquad \Gamma \vdash N_1, N_2 : \beta}{\Gamma \vdash \mathbf{if}\, M\, \mathbf{then}\, N_1\, \mathbf{else}\, N_2 : \beta} \qquad \frac{\Gamma \vdash M : \mathbf{exp} \qquad \Gamma \vdash N : \mathbf{com}}{\Gamma \vdash \mathbf{while}\, M\, \mathbf{do}\, N : \mathbf{com}}$$

$$\frac{}{\Gamma, x : \theta \vdash x : \theta} \qquad \frac{\Gamma, x : \theta \vdash M : \theta'}{\Gamma \vdash \lambda x.M : \theta \to \theta'} \qquad \frac{\Gamma \vdash M : \theta \to \theta' \qquad \Gamma \vdash N : \theta}{\Gamma \vdash MN : \theta'}$$

$$\frac{\Gamma \vdash M : \mathbf{var} \qquad \Gamma \vdash N : \mathbf{exp}}{\Gamma \vdash M := N : \mathbf{com}} \qquad \frac{\Gamma \vdash M : \mathbf{var}}{\Gamma \vdash !M : \mathbf{exp}}$$

$$\frac{\Gamma \vdash M : \mathbf{sem}}{\Gamma \vdash \mathbf{release}(M) : \mathbf{com}} \qquad \frac{\Gamma \vdash M : \mathbf{sem}}{\Gamma \vdash \mathbf{grab}(M) : \mathbf{com}}$$

$$\frac{\Gamma, x : \mathbf{var} \vdash M : \mathbf{com}, \mathbf{exp}}{\Gamma \vdash \mathbf{newvar}\, x := i\, \mathbf{in}\, M : \mathbf{com}, \mathbf{exp}} \qquad \frac{\Gamma, x : \mathbf{sem} \vdash M : \mathbf{com}, \mathbf{exp}}{\Gamma \vdash \mathbf{newsem}\, x := i\, \mathbf{in}\, M : \mathbf{com}, \mathbf{exp}}$$

Fig. 1: FICA typing rules where **com** is the type of commands; **exp** that of $\{0, \ldots, max\}$-valued expressions; **var** that of assignable variables; and **sem** that of semaphores. The typing judgments are displayed in Figure 1. **skip** and $\mathbf{div}_\theta$ are constants representing termination and divergence respectively, $i$ ranges over $\{0, \cdots, max\}$, and **op** represents unary arithmetic operations, such as successor or predecessor (since we work over a finite datatype, operations of bigger arity can be defined using conditionals). Variables and semaphores can be declared locally via **newvar** and **newsem**. Variables are dereferenced using $!M$, and semaphores are manipulated using two (blocking) primitives, $\mathbf{grab}(s)$ and $\mathbf{release}(s)$, which grab and release the semaphore respectively. The small-step operational semantics of FICA is reproduced in the full paper [18, Appendix A]. We shall write **div** for $\mathbf{div_{com}}$.

We are interested in *contextual equivalence* of terms. Two terms are contextually equivalent if there is no context that can distinguish them with respect to may-termination. More formally, a term $\vdash M : \mathbf{com}$ is said to terminate, written $M \Downarrow$, if there exists a terminating evaluation sequence from $M$ to **skip**. Then *contextual (may-)equivalence* ($\Gamma \vdash M_1 \cong M_2$) is defined by: for all contexts $\mathcal{C}$ such that $\vdash \mathcal{C}[M] : \mathbf{com}$, $\mathcal{C}[M_1] \Downarrow$ if and only if $\mathcal{C}[M_2] \Downarrow$. The force of this notion is quantification over all contexts.

Since contextual equivalence becomes undecidable for FICA very quickly [23], we will look at the special case of testing equivalence with terms that always diverge, e.g. given $\Gamma \vdash M : \theta$, is it the case that $\Gamma \vdash M \cong \mathbf{div}_\theta$? Intuitively, equivalence with an always-divergent term means that $\mathcal{C}[M]$ will never converge (must diverge) if $\mathcal{C}$ uses $M$. At the level of automata, this will turn out to correspond to the emptiness problem.

In verification tasks, with the above equivalence test, we can check whether uses of $M$ can ever lead to undesirable states. For example, for a given term $x : \mathbf{var} \vdash M : \theta$, the term

$$f : \theta \rightarrow \mathbf{com} \vdash \mathbf{newvar}\, x := 0\, \mathbf{in}\, (f(M) \,||\, \mathbf{if}\, !x = 13\, \mathbf{then\ skip\ else\ div})$$

will be equivalent to **div** only when $x$ is never set to 13 during a terminating execution. Note that, because of quantification over all contexts, $f$ may use $M$ an arbitrary number of times, also concurrently or in nested fashion, which is a very expressive form of quantification.

## 3 Game semantics

Game semantics for programming languages involves two players, called Opponent (O) and Proponent (P), and the sequences of moves made by them can be viewed as interactions between a program (P) and a surrounding context (O). In this section, we briefly present the fully abstract game model for FICA from [22], which we rely on in the paper. The games are defined using an auxiliary concept of an arena.

**Definition 1.** *An* arena *$A$ is a triple $\langle M_A, \lambda_A, \vdash_A \rangle$ where:*

- *$M_A$ is a set of* moves*;*
- *$\lambda_A : M_A \rightarrow \{O, P\} \times \{Q, A\}$ is a function determining for each $m \in M_A$ whether it is an* Opponent *or a* Proponent move*, and a* question *or an* answer*; we write $\lambda_A^{OP}, \lambda_A^{QA}$ for the composite of $\lambda_A$ with respectively the first and second projections;*
- *$\vdash_A$ is a binary relation on $M_A$, called* enabling*, satisfying: if $m \vdash_A n$ for no $m$ then $\lambda_A(n) = (O, Q)$, if $m \vdash_A n$ then $\lambda_A^{OP}(m) \neq \lambda_A^{OP}(n)$, and if $m \vdash_A n$ then $\lambda_A^{QA}(m) = Q$.*

We shall write $I_A$ for the set of all moves of $A$ which have no enabler; such moves are called *initial*. Note that an initial move must be an Opponent question. In arenas used to interpret base types all questions are initial and P-moves answering them are detailed in the table below, where $i \in \{0, \cdots, max\}$.

| Arena | O-question | P-answers | Arena | O-question | P-answers |
|---|---|---|---|---|---|
| $[\![\mathbf{com}]\!]$ | run | done | $[\![\mathbf{exp}]\!]$ | q | $i$ |
| $[\![\mathbf{var}]\!]$ | read<br>write($i$) | $i$<br>ok | $[\![\mathbf{sem}]\!]$ | grb<br>rls | ok<br>ok |

More complicated types are interpreted inductively using the *product* $(A \times B)$ and *arrow* $(A \Rightarrow B)$ constructions, given below.

$$\begin{array}{ll} M_{A\times B} = M_A + M_B & M_{A\Rightarrow B} = M_A + M_B \\ \lambda_{A\times B} = [\lambda_A, \lambda_B] & \lambda_{A\Rightarrow B} = [\langle \lambda_A^{PO}, \lambda_A^{QA} \rangle, \lambda_B] \\ \vdash_{A\times B} = \vdash_A + \vdash_B & \vdash_{A\Rightarrow B} = \vdash_A + \vdash_B + \{\, (b,a) \mid b \in I_B \text{ and } a \in I_A \} \end{array}$$

where $\lambda_A^{PO}(m) = O$ iff $\lambda_A^{OP}(m) = P$. We write $[\![\theta]\!]$ for the arena corresponding to type $\theta$. Below we draw (the enabling relations of) $A_1 = [\![\mathbf{com} \to \mathbf{com} \to \mathbf{com}]\!]$ and $A_2 = [\![(\mathbf{var} \to \mathbf{com}) \to \mathbf{com}]\!]$ respectively, using superscripts to distinguish copies of the same move (the use of superscripts is consistent with our future use of tags in Definition 9).

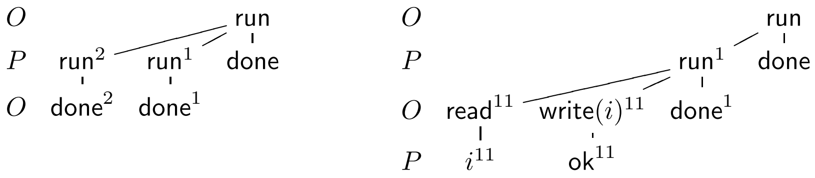

Given an arena $A$, we specify next what it means to be a legal play in $A$. For a start, the moves that players exchange will have to form a *justified sequence*, which is a finite sequence of moves of $A$ equipped with pointers. Its first move is always initial and has no pointer, but each subsequent move $n$ must have a unique pointer to an earlier occurrence of a move $m$ such that $m \vdash_A n$. We say that $n$ is (explicitly) justified by $m$ or, when $n$ is an answer, that $n$ answers $m$. If a question does not have an answer in a justified sequence, we say that it is *pending* in that sequence. Below we give two justified sequences from $A_1$ and $A_2$ respectively.

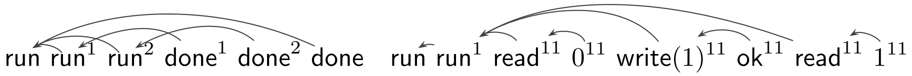

Not all justified sequences are valid. In order to constitute a legal play, a justified sequence must satisfy a well-formedness condition that reflects the "static" style of concurrency of our programming language: any started sub-processes must end before the parent process terminates. This is formalised as follows, where the letters $q$ and $a$ to refer to question- and answer-moves respectively, while $m$ denotes arbitrary moves.

**Definition 2.** *The set $P_A$ of* plays over $A$ *consists of the justified sequences $s$ over $A$ that satisfy the two conditions below.*

**FORK** *: In any prefix $s' = \cdots q \cdots m$ of $s$, the question $q$ must be pending when $m$ is played.*

**WAIT** *: In any prefix $s' = \cdots q \cdots a$ of $s$, all questions justified by $q$ must be answered.*

It is easy to check that the justified sequences given above are plays. A subset $\sigma$ of $P_A$ is *O-complete* if $s \in \sigma$ and $so \in P_A$ imply $so \in \sigma$, when $o$ is an O-move.

**Definition 3.** *A* strategy *on $A$, written $\sigma : A$, is a prefix-closed O-complete subset of $P_A$.*

Suppose $\Gamma = \{x_1 : \theta_1, \cdots, x_l : \theta_l\}$ and $\Gamma \vdash M : \theta$ is a FICA-term. Let us write $[\![\Gamma \vdash \theta]\!]$ for the arena $[\![\theta_1]\!] \times \cdots \times [\![\theta_l]\!] \Rightarrow [\![\theta]\!]$. In [22] it is shown how to assign a strategy on $[\![\Gamma \vdash \theta]\!]$ to any $\mathsf{FICA}$-term $\Gamma \vdash M : \theta$. We write $[\![\Gamma \vdash M]\!]$ to refer to that strategy. For example, $[\![\Gamma \vdash \mathbf{div}]\!] = \{\epsilon, \mathsf{run}\}$ and $[\![\Gamma \vdash \mathbf{skip}]\!] = \{\epsilon, \mathsf{run}, \overleftarrow{\mathsf{run}\,\mathsf{done}}\}$. Given a strategy $\sigma$, we denote by $\mathsf{comp}(\sigma)$ the set of non-empty *complete* plays of $\sigma$, i.e. those in which all questions have been answered. The game-semantic interpretation $[\![\cdots]\!]$ turns out to provide a fully abstract model in the following sense.

**Theorem 1 ([22]).** *$\Gamma \vdash M_1 \cong M_2$ iff $\mathsf{comp}([\![\Gamma \vdash M_1]\!]) = \mathsf{comp}([\![\Gamma \vdash M_2]\!])$.*

In particular, since we have $\mathsf{comp}([\![\Gamma \vdash \mathbf{div}_\theta]\!]) = \emptyset$, $\Gamma \vdash M : \theta$ is equivalent to $\mathbf{div}_\theta$ iff $\mathsf{comp}([\![\Gamma \vdash M]\!]) = \emptyset$.

## 4 Leafy automata

We would like to be able to represent the game semantics of $\mathsf{FICA}$ using automata. To that end, we introduce *leafy automata* ($\mathsf{LA}$). They are a variant of automata over nested data, i.e. a type of automata that read finite sequences of letters of the form $(t, d_0 d_1 \cdots d_j)$ ($j \in \mathbb{N}$), where $t$ is a *tag* from a finite set $\Sigma$ and each $d_i$ ($0 \leq i \leq j$) is a *data value* from an infinite set $\mathcal{D}$.

In our case, $\mathcal{D}$ will have the structure of a countably infinite forest and the sequences $d_0 \cdots d_j$ will correspond to branches of a tree. Thus, instead of $d_0 \cdots d_j$, we can simply write $d_j$, because $d_j$ uniquely determines its ancestors: $d_0, \ldots, d_{j-1}$. The following definition captures the technical assumptions on $\mathcal{D}$.

**Definition 4.** *$\mathcal{D}$ is a countably infinite set equipped with a function $pred : \mathcal{D} \to \mathcal{D} \cup \{\bot\}$ (the* parent *function) such that the following conditions hold.*

- *Infinite branching: $pred^{-1}(\{d_\bot\})$ is infinite for any $d_\bot \in \mathcal{D} \cup \{\bot\}$.*
- *Well-foundedness: for any $d \in \mathcal{D}$, there exists $i \in \mathbb{N}$, called the* level *of $d$, such that $pred^{i+1}(d) = \bot$. Level-0 data values will be called* roots.

In order to define configurations of leafy automata, we will rely on finite subtrees of $\mathcal{D}$, whose nodes will be labelled with states. We say that $T \subseteq \mathcal{D}$ is a subtree of $\mathcal{D}$ iff $T$ is closed ($\forall x \in T\colon pred(x) \in T \cup \{\bot\}$) and rooted ($\exists! x \in T\colon pred(x) = \bot$).

Next we give the formal definition of a level-$k$ leafy automaton. Its set of states $Q$ will be divided into layers, written $Q^{(i)}$ ($0 \leq i \leq k$), which will be used to label level-$i$ nodes. We will write $Q^{(i_1, \cdots, i_k)}$ to abbreviate $Q^{(i_1)} \times \cdots \times Q^{(i_k)}$, excluding any components $Q^{(i_j)}$ where $i_j < 0$. We distinguish $Q^{(0,-1)} = \{\dagger\}$.

**Definition 5.** *A level-$k$ leafy automaton ($k$-$\mathsf{LA}$) is a tuple $\mathcal{A} = \langle \Sigma, k, Q, \delta \rangle$, where*

- *$\Sigma = \Sigma_{\mathsf{Q}} + \Sigma_{\mathsf{A}}$ is a finite alphabet, partitioned into questions and answers;*
- *$k \geq 0$ is the level parameter;*
- *$Q = \sum_{i=0}^{k} Q^{(i)}$ is a finite set of states, partitioned into sets $Q^{(i)}$ of level-$i$ states;*
- *$\delta = \delta_{\mathsf{Q}} + \delta_{\mathsf{A}}$ is a finite transition function, partitioned into question- and answer-related transitions;*

– $\delta_{\mathsf{Q}} = \sum_{i=0}^{k} \delta_{\mathsf{Q}}^{(i)}$, *where* $\delta_{\mathsf{Q}}^{(i)} \subseteq Q^{(0,1,\cdots,i-1)} \times \Sigma_{\mathsf{Q}} \times Q^{(0,1,\cdots,i)}$ *for* $0 \le i \le k$*;*
– $\delta_{\mathsf{A}} = \sum_{i=0}^{k} \delta_{\mathsf{A}}^{(i)}$, *where* $\delta_{\mathsf{A}}^{(i)} \subseteq Q^{(0,1,\cdots,i)} \times \Sigma_{\mathsf{A}} \times Q^{(0,1,\cdots,i-1)}$ *for* $0 \le i \le k$*.*

Configurations of $\mathsf{LA}$ are of the form $(D, E, f)$, where $D$ is a finite subset of $\mathcal{D}$ (consisting of data values that have been encountered so far), $E$ is a finite subtree of $\mathcal{D}$, and $f : E \to Q$ is a level-preserving function, i.e. if $d$ is a level-$i$ data value then $f(d) \in Q^{(i)}$. A leafy automaton starts from the empty configuration $\kappa_0 = (\emptyset, \emptyset, \emptyset)$ and proceeds according to $\delta$, making two kinds of transitions. Each kind manipulates a single leaf: for questions one new leaf is added, for answers one leaf is removed. Let the current configuration be $\kappa = (D, E, f)$.

– On reading a letter $(t, d)$ with $t \in \Sigma_{\mathsf{Q}}$ and $d \notin D$ a fresh level-$i$ data, the automaton adds a new leaf $d$ in a configuration and updates the states on the branch to $d$. So it changes its configuration to $\kappa' = (D \cup \{d\}, E \cup \{d\}, f')$ provided that $\mathit{pred}(d) \in E$ and $f'$ satisfies:

$$(f(\mathit{pred}^i(d)), \cdots, f(\mathit{pred}(d)), t, f'(\mathit{pred}^i(d)), \cdots, f'(\mathit{pred}(d)), f'(d)) \in \delta_{\mathsf{Q}}^{(i)},$$

$\mathsf{dom}(f') = \mathsf{dom}(f) \cup \{d\}$, and $f'(x) = f(x)$ for all $x \notin \{\mathit{pred}(d), \cdots, \mathit{pred}^i(d)\}$.
– On reading a letter $(t, d)$ with $t \in \Sigma_{\mathsf{A}}$ and $d \in E$ a level-$i$ data which is a leaf, the automaton deletes $d$ and updates the states on the branch to $d$. So it changes its configuration to $\kappa' = (D, E \setminus \{d\}, f')$ where $f'$ satisfies:

$$(f(\mathit{pred}^i(d)), \cdots, f(\mathit{pred}(d)), f(d), t, f'(\mathit{pred}^i(d)), \cdots, f'(\mathit{pred}(d))) \in \delta_{\mathsf{A}}^{(i)},$$

$\mathsf{dom}(f') = \mathsf{dom}(f) \setminus \{d\}$ and $f'(x) = f(x)$ for all $x \notin \{\mathit{pred}(d), \cdots, \mathit{pred}^i(d)\}$.
– Initially $D$,$E$, and $f$ are empty; we proceed to $\kappa' = (\{d\}, \{d\}, \{d \mapsto q^{(0)}\})$ if $(t, d)$ is read where $\dagger \xrightarrow{t} q^{(0)} \in \delta_{\mathsf{Q}}^{(0)}$. The last move is treated symmetrically.

In all cases, we write $\kappa \xrightarrow{(t,d)} \kappa'$. Note that a single transition can only change states on the branch ending in $d$. Other parts of the tree remain unchanged.

*Example 1.* Below we illustrate the effect of $\mathsf{LA}$ transitions. Let $D_1 = \{d_0, d_1, d_1'\}$ and $d_2 \notin D_1$. Let $\kappa_1 = (D_1, E_1, f_1)$, $\kappa_2 = (D_1 \cup \{d_2\}, E_2, f_2)$, $\kappa_3 = (D_1 \cup \{d_2\}, E_1, f_1)$, where the trees $E_1, E_2$ are displayed below and node annotations of the form $(q)$ correspond to values of $f_1, f_2$, e.g. $f_1(d_0) = q^{(0)}$.

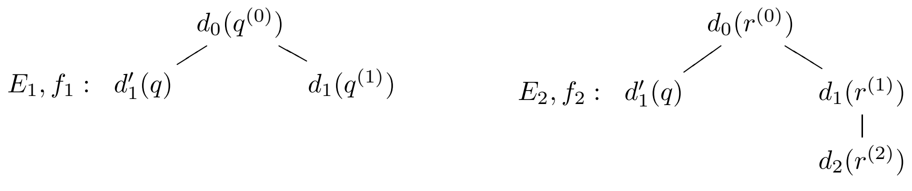

For $\kappa_1$ to evolve into $\kappa_2$ (on $(t, d_2)$), we need $(q^{(0)}, q^{(1)}, t, r^{(0)}, r^{(1)}, r^{(2)}) \in \delta_{\mathsf{Q}}^{(2)}$. On the other hand, to go from $\kappa_2$ to $\kappa_3$ (on $(t, d_2)$), we want $(r^{(0)}$, $r^{(1)}$, $r^{(2)}$, $t$, $q^{(0)}$, $q^{(1)}) \in \delta_{\mathsf{A}}^{(2)}$.

**Definition 6.** *A* trace *of a leafy automaton $\mathcal{A}$ is a sequence $w = l_1 \cdots l_h \in (\Sigma \times \mathcal{D})^*$ such that $\kappa_0 \xrightarrow{l_1} \kappa_1 \ldots \kappa_{h-1} \xrightarrow{l_h} \kappa_h$ where $\kappa_0 = (\emptyset, \emptyset, \emptyset)$. A configuration $\kappa = (D, E, f)$ is* accepting *if $E$ and $f$ are empty. A trace $w$ is accepted by $\mathcal{A}$ if there is a non-empty sequence of transitions as above with $\kappa_h$ accepting. The set of traces (resp. accepted traces) of $\mathcal{A}$ is denoted by $Tr(\mathcal{A})$ (resp. $L(\mathcal{A})$).*

*Remark 1.* When writing states, we will often use superscripts $(i)$ to indicate the intended level. So $(q^{(0)}, \cdots, q^{(i-1)}) \xrightarrow{t} (r^{(0)}, \cdots, r^{(i)})$ refers to $(q^{(0)}, \cdots, q^{(i-1)}, t, r^{(0)}, \cdots, r^{(i)}) \in \delta_{\mathsf{Q}}^{(i)}$; similarly for $\delta_{\mathsf{A}}^{(i)}$ transitions. For $i = 0$, this degenerates to $\dagger \xrightarrow{t} r^{(0)}$ and $r^{(0)} \xrightarrow{t} \dagger$.

*Example 2.* Consider the 1-LA over $\Sigma_{\mathsf{Q}} = \{\mathsf{start}, \mathsf{inc}\}, \Sigma_{\mathsf{A}} = \{\mathsf{dec}, \mathsf{end}\}$. Let $Q^{(0)} = \{0\}$, $Q^{(1)} = \{0\}$ and define $\delta$ by: $\dagger \xrightarrow{\mathsf{start}} 0$, $0 \xrightarrow{\mathsf{inc}} (0, 0)$, $(0, 0) \xrightarrow{\mathsf{dec}} 0$, $0 \xrightarrow{\mathsf{end}} \dagger$. The accepted traces of this 1-LA have the form $(\mathsf{start}, d_0)\ (||_{i=0}^{n} (\mathsf{inc}, d_1^i)\ (\mathsf{dec}, d_1^i))\ (\mathsf{end}, d_0)$, i.e. they are valid histories of a single non-negative counter (histories such that the counter starts and ends at 0). In this case, all traces are simply prefixes of such words.

*Remark 2.* Note that, whenever a leafy automaton reads $(t, d)$ $(t \in \Sigma_{\mathsf{Q}})$ and the level of $d$ is greater than 0, then it must have read a unique question $(t', pred(d))$ earlier. Also, observe that an LA trace contains at most two occurrences of the same data value, such that the first is paired with a question and the second is paired with an answer. Because the question and the answer share the same data value, we can think of the answer as answering the question, like in game semantics. Indeed, justification pointers from answers to questions will be represented in this way in Theorem 3. Finally, we note that LA traces are invariant under tree automorphisms of $\mathcal{D}$.

**Lemma 1.** *The emptiness problem for* 2-LA *is undecidable. For* 1-LA*, it is reducible to the reachability problem for VASS in polynomial time and there is a reverse reduction in exponential time, so it is decidable in Ackermannian time [32] but not elementary [13].*

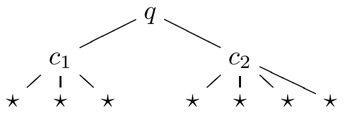

*Proof.* For 2-LA we reduce from the halting problem on two-counter-machines. Two counters can be simulated using configurations of the form where there are two level-1 nodes, one for each counter. The number of children at level 2 encodes the counter value. Zero tests can be implemented by removing the corresponding level-1 node and creating a new one. This is possible only when the node is a leaf, i.e., it does not have children at level 2. The state of the 2-counter machine can be maintained at level 0, the states at level 1 indicate the name of the counter, and the level-2 states are irrelevant.

The translation from 1-LA to VASS is straightforward and based on representing 1-LA configurations by the state at level 0 and, for each state at level 1, the count of its occurrences. The reverse translation is based on the same idea and extends the encoding of a non-negative counter in Example 2, where the exponential blow up is simply due to the fact that vector updates in VASS are given in binary whereas 1-LA transitions operate on single branches. □

**Lemma 2.** 1-LA *equivalence is undecidable.*

*Proof.* We provide a direct reduction from the halting problem for 2-counter machines, where both counters are required to be zero initially as well as finally. The main obstacle is that implementing zero tests as in the proof of the first part of Lemma 1 is not available because we are restricted to leafy automata with levels 0 and 1 only. To overcome it, we exploit the power of the equivalence problem where one of the 1-LA will have the task not of correctly simulating zero tests but recognising zero tests that are incorrect. The complete argument can be found in the full paper [18, Appendix B]. □

## 5 Local leafy automata (LLA)

Here we identify a restricted variant of LA for which the emptiness problem is decidable. We start with a technical definition.

**Definition 7.** *A $k$-LA is* bounded *at level $i$ ($0 \leq i \leq k$) if there is a bound $b$ such that each node at level $i$ can create at most $b$ children during a run. We refer to $b$ as the* branching bound*.*

Note that we are defining a "global" bound on the number of children that a node at level $i$ may create across a whole run, rather than a "local" bound on the number of children a node may have in a given configuration.

To motivate the design of LLA, we observe that the undecidability argument (for the emptiness problem) for 2-LA used two consecutive levels (0 and 1) that are not bounded. For the node at level 0, this corresponded to the number of zero tests, while an unbounded counter is simulated at level 1. In the following we will eliminate consecutive unbounded levels by introducing an alternating pattern of bounded and unbounded levels. Even-numbered layers ($i = 0, 2, ...$) will be bounded, while odd-numbered layers will be unbounded. Observe in particular that the root (layer 0) is bounded. As we will see later, this alternation reflects the term/context distinction in game semantics: the levels corresponding to terms are bounded, and the levels coresponding to contexts are unbounded.

With this restriction alone, it is possible to reconstruct the undecidability argument for 4-LA, as two unbounded levels may still communicate. Thus we introduce a restriction on how many levels a transition can read and modify.

– when adding or removing a leaf at an odd level $2i + 1$, the automaton will be able to access levels $2i$, $2i - 1$ and $2i - 2$; while

– when adding or removing a leaf at an even level $2i$, the automaton will be able to access levels $2i-1$ and $2i-2$.

In particular, when an odd level produces a leaf, it will not be able to see the previous odd level. The above constraints mean that the transition functions $\delta_{\mathsf{Q}}^{(i)}, \delta_{\mathsf{Q}}^{(i)}$ can be presented in a more concise form, given below.

$$\delta_{\mathsf{Q}}^{(i)} \subseteq \begin{cases} Q^{(i-2,i-1)} \times \Sigma_{\mathsf{Q}} \times Q^{(i-2,i-1,i)} & \text{if } i \text{ is even} \\ Q^{(i-3,i-2,i-1)} \times \Sigma_{\mathsf{Q}} \times Q^{(i-3,i-2,i-1,i)} & \text{if } i \text{ is odd} \end{cases}$$

$$\delta_{\mathsf{A}}^{(i)} \subseteq \begin{cases} Q^{(i-2,i-1,i)} \times \Sigma_{\mathsf{A}} \times Q^{(i-2,i-1)} & \text{if } i \text{ is even} \\ Q^{(i-3,i-2,i-1,i)} \times \Sigma_{\mathsf{A}} \times Q^{(i-3,i-2,i-1)} & \text{if } i \text{ is odd} \end{cases}$$

In terms of the previous notation used for $\mathsf{LA}$, $(q^{(i-2)}, q^{(i-1)}, x, r^{(i-2)}, r^{(i-1)}, r^{(i)}) \in \delta_{\mathsf{Q}}^{(i)}$ denotes all tuples of the form $(\vec{q}, q^{(i-2)}, q^{(i-1)}, x, \vec{q}, r^{(i-2)}, r^{(i-1)}, r^{(i)})$, where $\vec{q}$ ranges over $Q^{(0,\cdots,i-3)}$.

**Definition 8.** *A level-$k$* local leafy automaton *($k$-$\mathsf{LLA}$) is a $k$-$\mathsf{LA}$ whose transition function admits the above-mentioned presentation and which is bounded at all even levels.*

**Theorem 2.** *The emptiness problem for* $\mathsf{LLA}$ *is decidable.*

*Proof (Sketch).* Let $b$ be a bound on the number of children created by each even node during a run.

The critical observation is that, once a node $d$ at even level $2i$ has been created, all subsequent actions of descendants of $d$ access (read and/or write) the states at levels $2i-1$ and $2i-2$ at most $2b$ times. The shape of the transition function dictates that this can happen only when child nodes at level $2i+1$ are added or removed. In addition, the locality property ensures that the automaton will never access levels $< 2i-2$ at the same time as node $d$ or its descendants.

We will make use of these facts to construct *summaries* for nodes on even levels which completely describe such a node's lifetime, from its creation as a leaf until its removal, and in between performing at most $2b$ reads-writes of the parent and grandparent states. A summary is a sequence quadruples of states: two pairs of states of levels $2i-2$ and $2i-1$. The first pair are the states we expect to find on these levels, while the second are the states to which we update these levels. Hence a summary at level $2i$ is a complete record of a valid sequence of read-writes and stateful changes during the lifetime of a node on level $2i$.

We proceed by induction and show how to calculate the complete set of summaries at level $2i$ given the complete set of summaries at level $2i+2$. We construct a program for deciding whether a given sequence is a summary at level $2i$. This program can be evaluated via Vector Addition Systems with States (VASS). Since we can finitely enumerate all candidate summaries at level $2i$, this gives us a way to compute summaries at level $2i$. Proceeding this way, we finally calculate summaries at level 2. At this stage, we can reduce the emptiness problem for the given $\mathsf{LLA}$ to a reachability test on a VASS.

The complete argument is given in the full paper [18, Appendix C]. □

Let us remark also that the problem becomes undecidable if we remove either boundedness restriction, or allow transitions to look one level further.

## 6 From FICA to LA

Recall from Section 3 that, to interpret base types, game semantics uses moves from the set

$$\begin{aligned}\mathcal{M} &= M_{[\![\mathbf{com}]\!]} \cup M_{[\![\mathbf{exp}]\!]} \cup M_{[\![\mathbf{var}]\!]} \cup M_{[\![\mathbf{sem}]\!]}\\ &= \{\,\mathsf{run},\ \mathsf{done},\ \mathsf{q},\ \mathsf{read},\ \mathsf{grb},\ \mathsf{rls},\ \mathsf{ok}\,\} \cup \{\,i,\ \mathsf{write}(i)\,|\,0 \le i \le \max\,\}.\end{aligned}$$

The game semantic interpretation of a term-in-context $\Gamma \vdash M : \theta$ is a strategy over the arena $[\![\Gamma \vdash \theta]\!]$, which is obtained through product and arrow constructions, starting from arenas corresponding to base types. As both constructions rely on the disjoint sum, the moves from $[\![\Gamma \vdash \theta]\!]$ are derived from the base types present in types inside $\Gamma$ and $\theta$. To indicate the exact occurrence of a base type from which each move originates, we will annotate elements of $\mathcal{M}$ with a specially crafted scheme of superscripts. Suppose $\Gamma = \{x_1 : \theta_1, \cdots, x_l : \theta_l\}$. The superscripts will have one of the two forms, where $\vec{i} \in \mathbb{N}^*$ and $\rho \in \mathbb{N}$:

- $(\vec{i}, \rho)$ will be used to represent moves from $\theta$;
- $(x_v\vec{i}, \rho)$ will be used to represent moves from $\theta_v$ $(1 \le v \le l)$.

The annotated moves will be written as $m^{(\vec{i},\rho)}$ or $m^{(x_v\vec{i},\rho)}$, where $m \in \mathcal{M}$. We will sometimes omit $\rho$ on the understanding that this represents $\rho = 0$. Similarly, when $\vec{i}$ is omitted, the intended value is $\epsilon$. Thus, $m$ stands for $m^{(\epsilon,0)}$.

The next definition explains how the $\vec{i}$ superscripts are linked to moves from $[\![\theta]\!]$. Given $X \subseteq \{m^{(\vec{i},\rho)}\,|\,\vec{i} \in \mathbb{N}^*, \rho \in \mathbb{N}\}$ and $y \in \mathbb{N} \cup \{x_1, \cdots, x_l\}$, we let $yX = \{m^{(y\vec{i},\rho)}\,|\,m^{(\vec{i},\rho)} \in X\}$.

**Definition 9.** *Given a type $\theta$, the corresponding alphabet $\mathcal{T}_\theta$ is defined as follows*

$$\begin{aligned}\mathcal{T}_\beta &= \{\,m^{(\epsilon,\rho)}\,|\,m \in M_{[\![\beta]\!]},\ \rho \in \mathbb{N}\,\} \qquad \beta = \mathbf{com}, \mathbf{exp}, \mathbf{var}, \mathbf{sem}\\ \mathcal{T}_{\theta_h \to \ldots \to \theta_1 \to \beta} &= \textstyle\bigcup_{u=1}^{h}(u\mathcal{T}_{\theta_u}) \cup \mathcal{T}_\beta\end{aligned}$$

*For $\Gamma = \{x_1 : \theta_1, \cdots, x_l : \theta_l\}$, the alphabet $\mathcal{T}_{\Gamma\vdash\theta}$ is defined to be $\mathcal{T}_{\Gamma\vdash\theta} = \bigcup_{v=1}^{l}(x_v\mathcal{T}_{\theta_v}) \cup \mathcal{T}_\theta$.*

*Example 3.* The alphabet $\mathcal{T}_{f:\mathbf{com}\to\mathbf{com},x:\mathbf{com}\vdash\mathbf{com}}$ is $\{\mathsf{run}^{(f1,\rho)}, \mathsf{done}^{(f1,\rho)}, \mathsf{run}^{(f,\rho)}, \mathsf{done}^{(f,\rho)}, \mathsf{run}^{(x,\rho)}, \mathsf{done}^{(x,\rho)}, \mathsf{run}^{(\epsilon,\rho)}, \mathsf{done}^{(\epsilon,\rho)}\,|\,\rho \in \mathbb{N}\}$.

To represent the game semantics of terms-in-context, of the form $\Gamma \vdash M : \theta$, we are going to use *finite subsets* of $\mathcal{T}_{\Gamma\vdash\theta}$ as alphabets in leafy automata. The subsets will be finite, because $\rho$ will be bounded. Note that $\mathcal{T}_\theta$ admits a natural partitioning into questions and answers, depending on whether the underlying move is a question or answer.

We will represent plays using data words in which the underpinning sequence of tags will come from an alphabet as defined above. Superscripts and data are used to represent justification pointers. Intuitively, we represent occurrences of questions with data values. Pointers from answers to questions just refer to these values. Pointers from questions use bounded indexing with the help of $\rho$.

Initial question-moves do not have a pointer and to represent such questions we simply use $\rho = 0$. For non-initial questions, we rely on the tree structure of $\mathcal{D}$ and use $\rho$ to indicate the ancestor of the currently read data value that we mean to point at. Consider a trace $w(t_i, d_i)$ ending in a non-initial question, where $d_i$ is a level-$i$ data value and $i > 0$. In our case, we will have $t_i \in \mathcal{T}_{\Gamma \vdash \theta}$, i.e. $t_i = m^{(\cdots,\rho)}$. By Remark 2, trace $w$ contains unique occurrences of questions $(t_0, d_0), \cdots, (t_{i-1}, d_{i-1})$ such that $pred(d_j) = d_{j-1}$ for $j = 1, \cdots, i$. The pointer from $(t_i, d_i)$ goes to one of these questions, and we use $\rho$ to represent the scenario in which the pointer goes to $(t_{i-(1+\rho)}, d_{i-(1+\rho)})$.

Pointers from answer-moves to question-moves are represented simply by using the same data value in both moves (in this case we use $\rho = 0$).

We will also use $\epsilon$-tags $\epsilon_{\mathsf{Q}}$ (question) and $\epsilon_{\mathsf{A}}$ (answer), which do not contribute moves to the represented play. Each $\epsilon_{\mathsf{Q}}$ will always be answered with $\epsilon_{\mathsf{A}}$. Note that the use of $\rho, \epsilon_{\mathsf{Q}}, \epsilon_{\mathsf{A}}$ means that several data words may represent the same play (see Examples 4, 6).

*Example 4.* Suppose $d_0 = pred(d_1), d_1 = pred(d_2) = pred(d_2'), d_2 = pred(d_3)$, and $d_2' = pred(d_3')$. Then the data word $(\mathsf{run}, d_0)\ (\mathsf{run}^f, d_1)\ (\mathsf{run}^{f1}, d_2)\ (\mathsf{run}^{f1}, d_2')\ (\mathsf{run}^{(x,2)}, d_3)\ (\mathsf{run}^{(x,2)}, d_3')\ (\mathsf{done}^x, d_3)$, which is short for $(\mathsf{run}^{(\epsilon,0)}, d_0)\ (\mathsf{run}^{(f,0)}, d_1)\ (\mathsf{run}^{(f1,0)}, d_2)\ (\mathsf{run}^{(f1,0)}, d_2')\ (\mathsf{run}^{(x,2)}, d_3)\ (\mathsf{run}^{(x,2)}, d_3')\ (\mathsf{done}^{(x,0)}, d_3)$, represents the play

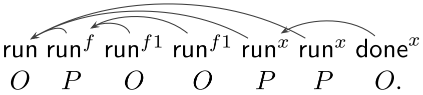

*Example 5.* Consider the LA $\mathcal{A} = \langle Q, 3, \Sigma, \delta \rangle$, where $Q^{(0)} = \{0, 1, 2\}$, $Q^{(1)} = \{0\}$, $Q^{(2)} = \{0, 1, 2\}$, $Q^{(3)} = \{0\}$, $\Sigma_{\mathsf{Q}} = \{\mathsf{run}, \mathsf{run}^f, \mathsf{run}^{f1}, \mathsf{run}^{(x,2)}\}$, $\Sigma_{\mathsf{A}} = \{\mathsf{done}, \mathsf{done}^f, \mathsf{done}^{f1}, \mathsf{done}^x\}$, and $\delta$ is given by

$$\dagger \xrightarrow{\mathsf{run}} 0 \qquad 0 \xrightarrow{\mathsf{run}^f} (1,0) \qquad (1,0) \xrightarrow{\mathsf{done}^f} 2 \qquad 2 \xrightarrow{\mathsf{done}} \dagger \qquad (1,0) \xrightarrow{\mathsf{run}^{f1}} (1,0,0)$$

$$(1,0,0) \xrightarrow{\mathsf{run}^{(x,2)}} (1,0,1,0) \qquad (1,0,1,0) \xrightarrow{\mathsf{done}^{(x,0)}} (1,0,2) \qquad (1,0,2) \xrightarrow{\mathsf{done}^{f1}} (1,0)$$

Then traces from $Tr(\mathcal{A})$ represent all plays from $\sigma = [\![ f : \mathbf{com} \to \mathbf{com}, x : \mathbf{com} \vdash fx ]\!]$, including the play from Example 4, and $L(\mathcal{A})$ represents $\mathsf{comp}(\sigma)$.

*Example 6.* One might wish to represent plays of $\sigma$ from the previous Example using data values $d_0, d_1, d_1', d_1'', d_2, d_2'$ such that $d_0 = pred(d_1) = pred(d_1') = pred(d_1'')$, $d_1 = pred(d_2) = pred(d_2')$, so that the play from Example 4 is represented by $(\mathsf{run}^{(\epsilon,0)}, d_0)\ (\mathsf{run}^{(f,0)}, d_1)\ (\mathsf{run}^{(f1,0)}, d_2)\ (\mathsf{run}^{(f1,0)}, d_2')\ (\mathsf{run}^{(x,0)}, d_1')\ (\mathsf{run}^{(x,0)}, d_1'')\ (\mathsf{done}^{(x,0)}, d_1')$. Unfortunately, it is impossible to construct a 2-LA that would accept all representations of such plays. To achieve this, the automaton would have to make sure that the number of $\mathsf{run}^{f1}$s is the same as that of $\mathsf{run}^x$s. Because the former are labelled with level-2 values and the latter with incomparable level-1 values, the only point of communication (that could be used for comparison) is the root. However, the root cannot accommodate unbounded information, while plays of $\sigma$ can feature an unbounded number of $\mathsf{run}^{f1}$s, which could well be consecutive.

Before we state the main result linking FICA with leafy automata, we note some structural properties of the automata. Questions will create a leaf, and answers will remove a leaf. P-moves add leaves at odd levels (questions) and remove leaves at even levels (answers), while O-moves have the opposite effect at each level. Finally, when removing nodes at even levels we will not need to check if a node is a leaf. We call the last property *even-readiness.*

Even-readiness is a consequence of the WAIT condition in the game semantics. The condition captures well-nestedness of concurrent interactions – a term can terminate only after subterms terminate. In the leafy automata setting, this is captured by the requirement that only leaf nodes can be removed, i.e. a node can be removed only if all of its children have been removed beforehand. It turns out that, for *P-answers* only, this property will come for free. Formally, whenever the automaton arrives at a configuration $\kappa = (D, E, f)$, where $d \in E$ and there is a transition

$$(f(pred^{(2i)}(d)), \cdots, f(pred(d)), f(d), t, f'(pred^{(2i)}(d)), \cdots, f'(pred(d))) \in \delta_{\mathsf{A}}^{(2i)},$$

then $d$ is a leaf. In contrast, our automata will not satisfy the same property for O-answers (the environment) and for such transitions it is crucial that the automaton actually checks that only leaves can be removed.

**Theorem 3.** *For any* FICA*-term $\Gamma \vdash M : \theta$, there exists an even-ready leafy automaton $\mathcal{A}_M$ over a finite subset of $\mathcal{T}_{\Gamma\vdash\theta} + \{\epsilon_{\mathsf{Q}}, \epsilon_{\mathsf{A}}\}$ such that the set of plays represented by data words from $Tr(\mathcal{A}_M)$ is exactly $[\![\Gamma \vdash M : \theta]\!]$. Moreover, $L(\mathcal{A}_M)$ represents $\mathsf{comp}([\![\Gamma \vdash M : \theta]\!])$ in the same sense.*

*Proof (Sketch).* Because every FICA-term can be converted to $\beta\eta$-normal form, we use induction on the structure of such normal forms. The base cases are: $\Gamma \vdash \mathbf{skip} : \mathbf{com}$ ($Q^{(0)} = \{0\}$; $\dagger \xrightarrow{\mathsf{run}} 0$, $0 \xrightarrow{\mathsf{done}} \dagger$), $\Gamma \vdash \mathbf{div} : \mathbf{com}$ ($Q^{(0)} = \{0\}$; $\dagger \xrightarrow{\mathsf{run}} 0$), and $\Gamma \vdash i : \mathbf{exp}$ ($Q^{(0)} = \{0\}$; $\dagger \xrightarrow{\mathsf{q}} 0$, $0 \xrightarrow{i} \dagger$).

The remaining cases are inductive. When referring to the inductive hypothesis for a subterm $M_i$, we shall use subscripts $i$ to refer to the automata components, e.g. $Q_i^{(j)}$, $\xrightarrow{\mathsf{m}}_i$ etc. In contrast, $Q^{(j)}$, $\xrightarrow{\mathsf{m}}$ will refer to the automaton that is being constructed. Inference lines —— will indicate that the transitions listed under the line should be added to the new automaton provided the transitions listed above the line are present in the automaton obtained via induction hypothesis. We discuss a selection of technical cases below.

$\Gamma \vdash M_1 || M_2$ In this case we need to run the automata for $M_1$ and $M_2$ concurrently. To this end, their level-0 states will be combined ($Q^{(0)} = Q_1^{(0)} \times Q_2^{(0)}$), but not deeper states ($Q^{(j)} = Q_1^{(j)} + Q_2^{(j)}, 1 \le j \le k$). The first group of transitions activate and terminate the two components respectively: $\frac{\dagger \xrightarrow{\mathsf{run}}_1 q_1^{(0)} \qquad \dagger \xrightarrow{\mathsf{run}}_2 q_2^{(0)}}{\dagger \xrightarrow{\mathsf{run}} (q_1^{(0)}, q_2^{(0)})}$,

$\frac{q_1^{(0)} \xrightarrow{\mathsf{done}}_1 \dagger \qquad q_2^{(0)} \xrightarrow{\mathsf{done}}_2 \dagger}{(q_1^{(0)},q_2^{(0)}) \xrightarrow{\mathsf{done}} \dagger}$. The remaining transitions advance each component:

$$\frac{(q_1^{(0)},\cdots,q_1^{(j)}) \xrightarrow{\mathsf{m}}_1 (r_1^{(0)},\cdots,r_1^{(j')}) \qquad q_2^{(0)} \in Q_2^{(0)}}{((q_1^{(0)},q_2^{(0)}),\cdots,q_1^{(j)}) \xrightarrow{\mathsf{m}} ((r_1^{(0)},q_2^{(0)}),\cdots,r_1^{(j')})} \quad \frac{q_1^{(0)} \in Q_1^{(0)} \qquad (q_2^{(0)},\cdots,q_2^{(j)}) \xrightarrow{\mathsf{m}}_2 (r_2^{(0)},\cdots,r_2^{(j')})}{((q_1^{(0)},q_2^{(0)}),\cdots,q_2^{(j)}) \xrightarrow{\mathsf{m}} ((q_1^{(0)},r_2^{(0)}),\cdots,r_2^{(j')})}$$

where $\mathsf{m} \neq \mathsf{run}, \mathsf{done}$.

$\Gamma \vdash \mathbf{newvar}\, x := i \,\mathbf{in}\, M_1$ By [22], the semantics of this term is obtained from the semantics of $[\![\Gamma, x \vdash M_1]\!]$ by

1. restricting to plays in which the moves $\mathsf{read}^x$, $\mathsf{write}(n)^x$ are followed immediately by answers,
2. selecting those plays in which each answer to a $\mathsf{read}^x$-move is consistent with the preceding $\mathsf{write}(n)^x$-move (or equal to $i$, if no $\mathsf{write}(n)^x$ was made),
3. erasing all moves related to $x$, e.g. those of the form $m^{(x,\rho)}$.

To implement 1., we will lock the automaton after each $\mathsf{read}^x$- or $\mathsf{write}(n)^x$-move, so that only an answer to that move can be played next. Technically, this will be done by adding an extra bit (lock) to the level-0 state. To deal with 2., we keep track of the current value of $x$, also at level 0. This makes it possible to ensure that answers to $\mathsf{read}^x$ are consistent with the stored value and that $\mathsf{write}(n)^x$ transitions cause the right change. Erasing from condition 3 is implemented by replacing all moves with the $x$ subscript with $\epsilon_{\mathsf{Q}}, \epsilon_{\mathsf{A}}$-tags.

Accordingly, we have $Q^{(0)} = (Q_1^{(0)} + (Q_1^{(0)} \times \{lock\})) \times \{0, \cdots, max\}$ and $Q^{(j)} = Q_1^{(j)}$ $(1 \leq j \leq k)$. As an example of a transition, we give the transition related to writing: $\frac{(q_1^{(0)},\cdots,q_1^{(j)}) \xrightarrow{\mathsf{write}(z)^{(x,\rho)}}_1 (r_1^{(0)},\cdots,r_1^{(j')}) \qquad 0 \leq n,z \leq max}{((q_1^{(0)},n),\cdots,q_1^{(j)}) \xrightarrow{\epsilon_{\mathsf{Q}}} ((r_1^{(0)},lock,z),\cdots,r_1^{(j')})}$.

$\Gamma \vdash f M_h \cdots M_1 : \mathbf{com}$ *with* $(f : \theta_h \to \cdots \to \theta_1 \to \mathbf{com})$ Here we will need $Q^{(0)} = \{0,1,2\}$, $Q^{(1)} = \{0\}$, $Q^{(j+2)} = \sum_{u=1}^{h} Q_u^{(j)}$ $(0 \leq j \leq k)$. The first group of transitions corresponding to calling and returning from $f$: $\dagger \xrightarrow{\mathsf{run}} 0$, $0 \xrightarrow{\mathsf{run}^f} (1,0)$, $(1,0) \xrightarrow{\mathsf{done}^f} 2$, $2 \xrightarrow{\mathsf{done}} \dagger$. Additionally, in state $(1,0)$ we want to enable the environment to spawn an unbounded number of copies of each of $\Gamma \vdash M_u : \theta_u$ $(1 \leq u \leq h)$. This is done through rules that embed the actions of the automata for $M_u$ while (possibly) relabelling the moves in line with our convention for representing moves from game semantics. Such transitions have the general form $\frac{(q_u^{(0)},\cdots,q_u^{(j)}) \xrightarrow{m^{(t,\rho)}}_u (q_u^{(0)},\cdots,q_u^{(j')})}{(1,0,q_u^{(0)},\cdots,q_u^{(j)}) \xrightarrow{m^{(t',\rho')}} (1,0,q_u^{(0)},\cdots,q_u^{(j')})}$. Note that this case also covers $f : \mathbf{com}$ $(h = 0)$.

More details and the remaining cases are covered in the full paper [18, Appendix D], along with an example of a term and the corresponding $\mathsf{LA}$. $\square$

## 7 Local FICA

In this section we identify a family of FICA terms that can be translated into LLA rather than LA. To achieve boundedness at even levels, we remove while[5]. To achieve restricted communication, we will constrain the distance between a variable declaration and its use. Note that in the translation, the application of function-type variables increases LA depth. So in LFICA we will allow the link between the binder **newvar**/**newsem** $x$ and each use of $x$ to "cross" at most one occurrence of a free variable. For example, the following terms

- $\mathbf{newvar}\, x := 0\, \mathbf{in}\, x := 1 \,||\, f(x := 2)$,
- $\mathbf{newvar}\, x := 0\, \mathbf{in}\, f(\mathbf{newvar}\, y\, \mathbf{in}\, f(y := 1) \,||\, x := !y)$

will be allowed, but not $\mathbf{newvar}\, x := 0\, \mathbf{in}\, f(f(x := 1))$.

To define the fragment formally, given a term $Q$ in $\beta\eta$-normal form, we use a notion of the *applicative depth of a variable* $x : \beta$ *(*$\beta = \mathbf{var}, \mathbf{sem}$*) inside* $Q$, written $ad_x(Q)$ and defined inductively by the table below. The applicative depth is increased whenever a functional identifier is applied to a term containing $x$.

| shape of $Q$ | $ad_x(Q)$ |
|---|---|
| $x$ | 1 |
| $y\,(y \neq x)$, **skip**, **div**, $i$ | 0 |
| $\mathbf{op}(M)$, $!M$, $\mathbf{release}(M)$, $\mathbf{grab}(M)$ | $ad_x(M)$ |
| $M; N$, $M \| N$, $M := N$, $\mathbf{while}\, M\, \mathbf{do}\, N$ | $\max(ad_x(M), ad_x(N))$ |
| $\mathbf{if}\, M\, \mathbf{then}\, N_1\, \mathbf{else}\, N_2$ | $\max(ad_x(M), ad_x(N_1), ad_x(N_2))$ |
| $\lambda y.M, \mathbf{newvar}\, /\mathbf{newsem}\, y := i\, \mathbf{in}\, M$ | $ad_x(M[z/y])$, where $z$ is fresh |
| $f M_1 \cdots M_k$ | $1 + \max(ad_x(M_1), \cdots, ad_x(M_k))$ |

Note that in our examples above, in the first two cases the applicative depth of $x$ is 2; and in the third case it is 3.

**Definition 10 (Local FICA).** *A FICA-term* $\Gamma \vdash M : \theta$ *is* local *if its* $\beta\eta$*-normal form does not contain any occurrences of* **while** *and, for every subterm of the normal form of the shape* $\mathbf{newvar}\, /\mathbf{newsem}\, x := i\, \mathbf{in}\, N$*, we have* $ad_x(N) \leq 2$*. We write* LFICA *for the set of local* FICA *terms.*

**Theorem 4.** *For any* LFICA*-term* $\Gamma \vdash M : \theta$*, the automaton* $\mathcal{A}_M$ *obtained from the translation in Theorem 3 can be presented as a* LLA*.*

*Proof (Sketch).* We argue by induction that the constructions from Theorem 3 preserve presentability as a LLA.

The case of parallel composition involves running copies of $M_1$ and $M_2$ in parallel without communication, with their root states stored as a pair at level 0. Note, though, that each of the automata transitions independently of the state of the other automaton. In consequence, if the automata $M_1$ and $M_2$ are LLA, so

[5] The automaton for $\mathbf{while}\, M\, \mathbf{do}\, N$ may repeatedly visit the automata for $M$ and $N$, generating an unbounded number of children at level 0 in the process.

will be the automaton for $M_1||M_2$. The branching bound after the construction is the sum of the two bounds for $M_1$ and $M_2$.

For $\Gamma \vdash \mathbf{newvar}\, x := i\, \mathbf{in}\, M$, because the term is in LFICA, so is $\Gamma, x : \mathbf{var} \vdash M$ and we have $ad_x(M) \leq 2$. Then we observe that in the translation of Theorem 3 ($\Gamma, x : \mathbf{var} \vdash M : \theta$) the questions related to $x$, (namely $\mathsf{write}(i)^{(x,\rho)}$ and $\mathsf{read}^{(x,\rho)}$) correspond to creating leaves at levels 1 or 3, while the corresponding answers ($\mathsf{ok}^{(x,\rho)}$ and $i^{(x,\rho)}$ respectively) correspond to removing such leaves. In the construction for $\Gamma \vdash \mathbf{newvar}\, x\, \mathbf{in}\, M$, such transitions need access to the root (to read/update the current state) and the root is indeed within the allowable range: in an LLA transitions creating/destroying leaves at level 3 can read/write at level 0. All other transitions (not labelled by $x$) proceed as in $M$ and need not consult the root for additional information about the current state, as it is propagated. Consequently, if $M$ is represented by a LLA then the interpretation of $\mathbf{newvar}\, x := i\, \mathbf{in}\, M$ is also a LLA. The construction does not affect the branching bound, because the resultant runs can be viewed as a subset of runs of the automaton for $M$, i.e. those in which reads and writes are related.

For $f M_h \cdots M_1$, we observe that the construction first creates two nodes at levels 0 and 1, and the node at level 1 is used to run an unbounded number of copies of (the automaton for) $M_i$. The copies do not need access to the states stored at levels 0 and 1, because they are never modified when the copies are running. Consequently, if each $M_i$ can be translated into a LLA, the outcome of the construction in Theorem 3 is also a LLA. The new branching bound is the maximum over bounds from $M_1, \cdots, M_h$, because at even levels children are produced as in $M_i$ and level 0 produces only 1 child. □

**Corollary 1.** *For any* LFICA*-term $\Gamma \vdash M : \theta$, the problem of determining whether $\mathsf{comp}([\![\Gamma \vdash M]\!])$ is empty is decidable.*

Theorems 1 and 2 imply the above. Thanks to Theorem 1, it is decidable if a LFICA term is equivalent to a term that always diverges (cf. example on page 187). In case of inequivalence, our results could also be applied to extract the distinguishing context, first by extracting the witnessing trace from the argument underpinning Theorem 2 and then feeding it to the Definability Theorem (Theorem 41 [22]). This is a valuable property given that in the concurrent setting bugs are difficult to replicate.

## 8 From LA to FICA

In this section, we show how to represent leafy automata in FICA. Let $\mathcal{A} = \langle \Sigma, k, Q, \delta \rangle$ be a leafy automaton. We shall assume that $\Sigma, Q \subseteq \{0, \cdots, max\}$ so that we can encode the alphabet and states using type **exp**. We will represent a trace $w$ generated by $\mathcal{A}$ by a play $\mathsf{play}(w)$, which simulates each transition with two moves, by $O$ and $P$ respectively. The child-parent links in $\mathcal{D}$ will be represented by justification pointers. We refer the reader to [18, Appendix F] for details. Below we just state the lemma that identifies the types that correspond to our encoding, where we write $\theta^{max+1} \to \beta$ for $\underbrace{\theta \to \cdots \to \theta}_{max+1} \to \beta$.

**Lemma 3.** *Let* $\mathcal{A}$ *be a* $k$-LA *and* $w \in Tr(\mathcal{A})$*. Then* $\mathsf{play}(w)$ *is a play in* $[\![\theta_k]\!]$*, where* $\theta_0 = \mathbf{com}^{max+1} \rightarrow \mathbf{exp}$ *and* $\theta_{i+1} = (\theta_i \rightarrow \mathbf{com})^{max+1} \rightarrow \mathbf{exp}$ *(*$i \geq 0$*).*

Before we state the main result, we recall from [22] that strategies corresponding to FICA terms satisfy a closure condition known as *saturation*: swapping two adjacent moves in a play belonging to such a strategy yields another play from the same strategy, as long as the swap yields a play and it is not the case that the first move is by O and the second one by P. Thus, saturated strategies express causal dependencies of P-moves on O-moves. Consequently, one cannot expect to find a FICA-term such that the corresponding strategy is the smallest strategy containing $\{\, \mathsf{play}(w) \mid w \in Tr(\mathcal{A}) \,\}$. Instead, the best one can aim for is the following result.

**Theorem 5.** *Given a* $k$-LA $\mathcal{A}$*, there exists a* FICA *term* $\vdash M_{\mathcal{A}} : \theta_k$ *such that* $[\![ \vdash M_{\mathcal{A}} : \theta_k ]\!]$ *is the smallest saturated strategy containing* $\{\, \mathsf{play}(w) \mid w \in Tr(\mathcal{A}) \,\}$*.*

*Proof (Sketch).* Our assumption $Q \subseteq \{0, \cdots, max\}$ allows us to maintain $\mathcal{A}$-states in the memory of FICA-terms. To achieve $k$-fold nesting, we use the higher-order structure of the term: $\lambda f^{(0)}.f^{(0)}(\lambda f^{(1)}.f^{(1)}(\lambda f^{(2)}.f^{(2)}(\cdots \lambda f^{(k)}.f^{(k)})))$. In fact, instead of the single variables $f^{(i)}$, we shall use sequences $f_0^{(i)} \cdots f_{max}^{(i)}$, so that a question $t_{\mathsf{Q}}^{(i)}$ read by $\mathcal{A}$ at level $i$ can be simulated by using variable $f_{t_{\mathsf{Q}}^{(i)}}^{(i)}$ (using our assumption $\Sigma \subseteq \{0, \cdots, max\}$). Additionally, the term contains state-manipulating code that enables moves only if they are consistent with the transition function of $\mathcal{A}$. □

## 9 Conclusion and further work

We have introduced leafy automata, LA, and shown that they correspond to the game semantics of Finitary Idealized Concurrent Algol (FICA). The automata formulation makes combinatorial challenges posed by the equivalence problem explicit. This is exemplified by a very transparent undecidability proof of the emptiness problem for LA. Our hope is that LA will allow to discover interesting fragments of FICA for which some variant of the equivalence problem is decidable. We have identified one such instance, namely local leafy automata (LLA), and a fragment of FICA that can be translated to them. The decidability of the emptiness problem for LLA implies decidability of a simple instance of the equivalence problem. This in turn allows to decide some verification questions as in the example on page 187. Since these types of questions involve quantification over all contexts, the use of a fully-abstract semantics appears essential to solve them.

The obvious line of future work is to find some other subclasses of LA with decidable emptiness problem. Another interesting target is to find an automaton model for the call-by-value setting, where answers enable questions [2,25]. It would also be worth comparing our results with abstract machines [19], the Geometry of Interaction [31], and the $\pi$-calculus [6].
